# Genotype-phenotype variable correlation in Wilson disease: clinical history of two sisters with the similar genotype

**DOI:** 10.1186/s12881-020-01062-6

**Published:** 2020-06-12

**Authors:** Annamaria Sapuppo, Piero Pavone, Andrea Domenico Praticò, Martino Ruggieri, Gaetano Bertino, Agata Fiumara

**Affiliations:** Department of Clinical and Experimental Medicine, University of Catania, Catania, Italy

**Keywords:** Wilson disease, Genotype-phenotype correlation, ATP7B gene - ceruloplasmin, Copper - hepatolenticular degeneration

## Abstract

**Background:**

Wilson disease (WD) is an Autosomal-Recessive disorder due to mutations of ***ATP7B*** gene on chromosome *13q14.3*. Inadequate protein function leads to low ceruloplasmin blood levels and copper accumulation in liver, basal ganglia and chornea. Main clinical manifestations are hypertransaminasemia, tremors, dysarthria, dystonia and psychiatric symptoms. The phenotypic variability in WD is considerable and its onset can be heterogeneous: the most common type in childhood is the hepatic involvement, followed by the neurological one or others. The presence of a genotype-phenotype correlation has not yet been fully demonstrated. The phenotypic variability may be explained by the intervention of other modifier genes regulating copper metabolism in the presence of mutations ***ATP7B.***

**Case presentation:**

A streaking phenotypic variability was observed in two Sicilian sisters carrying the same genotype for ***ATB7B*** gene [*c.3207C > A* / c.*3904-2A > G*]. Although both started to present signs at age 10 years, onset was characterized by neurological signs in the first (tremors, motor incoordination, language and cognitive impairment), while liver involvement has been the only sign in the other. They started the same chelation therapy. After a 20-year follow-up the former is severely affected (MRI evidence of basal ganglia copper deposits and hyperchogenic liver, thrombocytopenia), while the latter presents only a moderate liver enlargement. In literature, the splice mutation c.*3904-2A > G* is also reported in Egypt population, associated with acute liver failure or chronic hepatic disease, and it could be typical of Mediterranean area, not being reported in other geographical zones.

**Conclusion:**

Based on our clinical experience in Eastern Sicily, there is a considerable phenotypic variability in WD, even in the presence of an identical genotype. The mutation c.*3904-2A > G* could be associated with this phenotypic variability in Mediterranean population, but further studies should be conducted. This condition could be explained by the intervention of modifier genes regulating copper metabolism in the presence of defective ***ATP7B*** protein function. Further investigations on their role by Next Generation Sequencing or Whole Exome Analysis might have a profound impact on patients’ management and in particular on therapy.

## Background

Wilson disease (WD), also known as hepatolenticular degeneration, is an autosomal recessive inherited disorder resulting from abnormal copper metabolism. It is due to mutations of the gene *ATP7B*, positioned on the long arm of chromosome 13 (*13q14.3*). Presently, more than 300 pathogenetic variants causing the disorder have been recognized.

The product of this gene is a membrane protein with ATPase function, which carries copper in the secretory pathway. It enables physiologically copper excretion in the bile and its incorporation in the ceruloplasmin, responsible for its transport in the bloodstream [1, 2].

The current model suggests WD to be caused by the presence of two mutant pathogenic *ATP7B* alleles. Indeed, the majority of patients are homozygous or compound heterozygous for *ATP7B* pathogenic mutations [3, 4], although a number of WD families carry only one or no pathogenic *ATP7B* mutations [3, 5, 6]. Single mutation in the *ATP7B* gene, or no mutations at all, encompass about 10% of clinically and biochemically diagnosed WD patients [7].

Inactive mutations of the gene *ATP7B*, either in homozygosis or in compound heterozygosis, cause a decreased copper excretion and a consequently progressive accumulation of this metal in the liver, following by chronic active hepatitis, fibrosis, and cirrhosis. Then, the liver releases copper into the bloodstream, where is bound to ceruloplasmin. This free copper precipitates throughout the body, particularly in the central nervous system (CNS), affecting the basal ganglia, in particular putamen, and globus pallidus. These areas are involved in the coordination of movement and neurocognitive processes and their dysfunction is cause of behavioral dysregulation.

In WD, other involved organs are cornea, with the pathognomonic presence of Kayser-Fleischer (KF) ring, kidneys, joints and cardiac muscle with consequent impair of their physiological functions. Sometimes WD can onset with psychiatric disorders, such as depression, difficulty in concentrating, memory loss and reduced scholastic performance and with difficulties in writing, reading and speech. Another clinical presentation is hemolytic anemia: indeed, copper acts as pro-oxidant agent, by increasing production of free radicals highly reactive, particularly on the cell membranes [8]. This dysfunction causes a progressive consumption of their antioxidant systems which results in irreversible damages, until cellular apoptosis.

Thus, the disease can be suspected not only in the presence of a complex set of symptoms, but also with specific, single signs involving neurological or hepatic organs, or other parts of the body, including blood, kidneys and bones.

We report here two sisters affected by WD who presented similar genotype (composed heterozygote for the mutations *c.3207C > A* – which causes the aminoacid substitution *p.His1069Gln* – and c.*3904-2A > G*, which alters a splicing site), but showing a significant phenotypic variability since the onset of the disorder.

## Case presentation

About the family history, the parents are unrelated and healthy. At the time of the first gestation the ages of the patient’s mother and father were 22 and 27 years old respectively. The mother denied any alcohol intake or cigarettes smoking during the pregnancies. A first cousin is affected by WD with hepatic onset (hypertransaminasemia and hepatomegaly) in absence of neurological symptoms. The parents were not available for their genetic analysis.

### Patient n°1

The first patient is a woman, 37 years old, first born from unrelated parents, after normal pregnancy and natural childbirth. Her psychomotor development was normal and her clinical history in the first years of life was irrelevant.

At the age of 11 years, she was admitted to the Pediatric Clinic of University of Catania, Italy because of the appearance of speech disorder, awkwardness in movements, especially during her walking, tremor in her right hand with writing difficulties, progressively worsened, with poor scholastic performances, with deficit in learning and concentration.

At the initial physical examination, it was found a mild splenomegaly with spleen palpable about 1 cm from the costal arch. Moreover, acrocyanosis in upper and lower limbs was noted and her right lower limb appears a little shorter and hypertrophic than the contralateral one.

She performed blood tests (see Table 1), which showed: anemia, leukopenia, thrombocytopenia. Transaminases and other liver parameters were normal, as well renal function.

**Table 1:** *Laboratory blood sample test comparison between the two patients of our study*

	**PATIENTS**				
	**N°1**		**N°2**		
**BLOOD TEST**	**ONSET**	**PRESENT**	**ONSET**	**PRESENT**	**NORMAL**
**CERULOPLASMIN** (gr/dl)	0.08	0.07	0.14	0.14	> 0.2 (20)
**SERUM COPPER** (μg/dl)	48	13	40	39	80–150
**URINARY COPPER** (μg / 24 h)	160	320 (under Penicillamine)	120	250 (under Penicillamine)	< 40
**Hb** (gr/dl)	11.3	13.7	13	13.2	12–18
**RBC** (Nx10^6^)	3.9 × 10^6^	4.8 × 10^6^	4.3 × 10^6^	4.4 × 10^6^	4–5.5 × 10^6^
**WBC** (Nx10^3^)	2.5 × 10^3^	5.1 × 10^3^	5 × 10^3^	6 × 10^3^	4.8–10.8 × 10^3^
**PLT** (Nx10^3^)	68 × 10^3^	54 × 10^3^	242 × 10^3^	247 × 10^3^	130–400 × 10^3^
**ALT** (U/L)	33	32	96	151	10–35
**AST** (U/L)	29	27	51	78	10–35
**LDH** (U/L)	554	514	429	277	250–500
**PCE** (U/L)	7000	3500	8500	6000	5000–12,000
**CHOLESTEROL** (mg/dl)	152	178	207	280	130–200
**TRIGLYCERIDS** (mg/dl)	39	61	237	190	10–150

***LEGEND:Hb*Haemoglobin*; RBC*Red Blood Cells*; WBC*White Blood Cells*; PLT*Platelet*; ALT*ALanine aminotransferase*; AST*ASpartate Transaminase*; LDH*Lactate dehydrogenase*; PCE*PseudoCholinEsterase**

At Abdomen ultrasound, the liver appeared in its normal location, with normal size and shape; the spleen was in its normal location, but increased in volume, with uniform echo-structure; nothing in other abdominal organs. Bone marrow aspirate was carried out, in order to exclude hematologic disorders, resulted normal.

Because of WD suspicion, plasma ceruloplasmin and copper was performed, resulting both lower than normal. After then, the urine collection in 24 h was done, which showed an increase in urinary copper excretion.

An eye test with fundus oculi examination was carried and KF rings were found, consistently with WD suspected diagnosis.

Brain MRI was performed, showing the *presence of abnormal signal areas featured by symmetric hyperintensities in basal and putamen ganglia (as probably due to copper deposit) and degenerative-spongiosis microcavitary, which acts to lenticular nuclei*.

Liver biopsy was not performed because this organ did not seem involved, moving forward directly with genetic investigation of *ATP7B* gene mutations.

This investigation noticed a compound heterozygosis for the mutations ***c.3207C > A (p.His1069Gln)*** and ***c.3904-2A > G***, confirming the diagnosis of WD.

Chelating therapy with D-penicillammine (1000 mg/d; 20 mg/kg/d) and vitamin B6 (50 mg twice a week) was immediately started (at that time in Italy zinc acetate was not available), with progressive improvement of neurological symptoms (despite slight dysgraphia and dysarthria remained). KF rings disappeared, with a stable situation of hypochondriac organs monitored periodically.

She presented a pubertal developmental delay (menarche at 16 years old) and menstrual cycles were often irregular.

In the following 10 years a progressive liver disease has occurred with persistent thrombocytopenia and low cholinesterase blood levels, poor responsive to drug chelating treatment. The liver revealed an abnormal echo-structure with micronodular appearance, irregular morphology in the venous splenic-portal axis with development of collateral circulation at splenic and hilar site. The spleen had an increased volume but homogeneous echo-structure.

It was performed abdomen MRI that confirmed the clinical picture, with evidence of splenic cavernoma.

Control brain MRI showed the persistence of symmetric hyperintensities areas in basal and putamen ganglia and a low dilation of cerebrospinal fluid (CSF) spaces, ventricular chambers and of cranial vault subarachnoid spaces.

In the eye examination test bilateral KF rings was found.

She needed combined treatment (chelating drug plus acetate zinc), preceded by liver biopsy to measure the hepatic disease and fibrosis, which enabled to diagnose hepatic cirrhosis.

She had a healthy son at the age of 33 and the genetic screening test for WD is ongoing. In the last 2 years, she starts showing psychiatric signs, as depressive status, untreated pharmacologically for patient choice. She had progressive refusal of her illness, with low drug compliance to combined pharmacological treatment and progressive clinical worsening. She did not practice physical activity regularly and did not follow a diet with adequate nutrient intake, with frequent weight changes. Currently, she is affected by severe hepatic cirrhosis (Child-Pugh score: C13, Meld score: 24). A recently episode of portal-systemic encephalopathy is appeared. She was put on the liver transplant list and follows a lacto-vegetarian diet with reduced animal protein content to prevent such episodes.

### Patient n°2

The second patient is the second born sister of patient n°1, aged 33 years old. She was born after normal pregnancy, by natural childbirth. Her psychomotor development was normal and her clinical history in the first years of life was negative for relevant diseases.

At the age of 10 years, because of abdominal colic, blood samples were done. There was an evidence of mild hypertransaminasemia (see Table 1) and hypertriglyceridemia in a normal weight patient.

A painful hepatomegaly was found at physical examination. Ceruloplasmin and serum copper both resulted lower than normal values, with an increase in urinary copper excretion, confirming the suspicion of WD.

Abdomen ultrasound was performed and a hepatomegaly with slight homogeneous hyperechogenicity of the parenchyma was found; nothing about other abdominal organs. Eye examination test with fundus oculi and brain MRI were both normal. Genetic study for the gene ***ATP7B*** revealed a state of compound heterozygosis for the mutations *c.3207C > A* (p.*His1069Gln*) and *c.3904-2A > G*, like her elder sister.

Chelating therapy with D-penicillammine and vitamin B6 was started, with progressive clinical and normalization of transaminases and triglycerides, while at abdomen ultrasound the liver remained slightly increased in size above the normal with persistent homogeneous hyperechogenicity of the parenchyma in the absence of focal alterations.

In the following controls, brain MRI and eye examination test were always negative for copper accumulation.

In the last 5 years, the patient demonstrated a low compliance to the therapy, confirmed by slightly increased values of transaminases (see Table 1) and triglycerides, while stable abdominal ultrasound and brain MRI picture. Combined therapy with acetate zinc and D-penicillammine was prescribed, but she reports a poor tolerability to zinc therapy due to gastrointestinal side effects and difficulty in taking the drug away from meals due to its working activity. For these reasons, chelation therapy only was done in the last year with an improving of therapeutic compliance and blood tests, probably also due to the psychological involvement of contemporary worsening of her sister. Currently, she has a good lifestyle; practices regular physical activity 3 times a week and follows a Mediterranean-type diet. The main features of two patients are summarized in Table 2.

**Table 2:** *Genotype-Phenotype comparison between the two patients of our study*

PATIENTS	N°1	N°2
**SEX**	F	F
**CURRENT AGE**	37 years	33 years
**TYPE OF ONSET**	**NEUROLOGICAL**	**HEPATIC**
**GENOTYPE**	**c.3207C- > A(p.H1069Q) /** **c.3904-2A- > G**	**c.3207C- > A(p.H1069Q) /** **c.3904-2A- > G**
**AGE OF ONSET AND DIAGNOSIS**	11 years	10 years
**PHYSICAL EXAMINATION (ONSET)**	Tremors with difficulty in writing, motor incoordination, speech and poor scholastic performances	Hepatomegaly, latent jaundice
**BLOOD TESTS (ONSET)**	Low ceruloplasmin and copper, anemia, thrombocytopenia, low WBC e RBC, no hypertransaminasemia (see Table 1)	Low ceruloplasmin and copper, hypertransaminasemia, hypercholesterolemia (see Table 1)
**EYE EXAMINATION (ONSET)**	KF rings in both eyes	No KF rings
**ABDOMINAL US (ONSET)**	Normal liver. Spleen increased in volume, with homogeneous parenchyma and vessel dilatation.	Liver normal in shape and size, with homogenous hyperechogenic parenchyma. Normal spleen.
**BRAIN MRI (ONSET)**	Hyperintensity of the basal ganglia, especially the lenticular nuclei and the putamen	Normal
**LEIPZIG SCORE**	11	7
**BLOOD TESTS (PRESENT DAYS)**	Thrombocytopenia, low cholinesterase (see Table 1)	Hypertransaminasemia, Hypertriglyceridemia (see Table 1)
**ABDOMINAL US (PRESENT DAYS)**	Liver echostructure severely inhomogeneous (cirrhosis). Signs of portal hypertension, voluminous collateral circulation, splenomegaly.	Liver with dimensions slightly larger than normal. Echostructure homogeneous without focal alterations.
**BRAIN MRI** **(PRESENT DAYS)**	Persistent hyperintensity of the basal ganglia	Normal
**CLINICAL SITUATION**	Portal systemic encephalopathy	Liver parenchyma hyperecogeniticy

## Discussion and conclusions

WD is an autosomal recessive disorder with a clinically high variability in phenotype, due to alterations in carrying copper that accumulates in several organs, particularly in liver, basal ganglia and cornea. The disorder usually onset between the age of 6 and 40 years [9]; in childhood it usually manifests with a chronic hepatic dysfunction more or less severe, persistent transaminase elevation, hyperbilirubinemia to frank jaundice until the development of hepatic cirrhosis or an acute fulminant hepatitis with necessity of liver transplant.

Neurological symptoms are usually less frequent before the second decade of life [10] and are typically represented by an extrapyramidal symptomatology. The initial symptoms in some case are learning and cognitive disorders and attention deficit hyperactivity disorder (ADHD). Brain MRI in these patients generally displays hypointensity areas in T1 and hyperintensity areas in T2 in the basal ganglia, but signs of brain and/or cerebellar atrophy and/or ventricular dilation can be also found [11], as in the first patient of the present report.

The presence of KF rings is considered pathognomonic of WDand is always present in patients with neurological onset, as in case 1. On the other hand, patients with hepatic onset, as happened in case 2, uncommonly show the KF rings at onset.

Despite being a rare genetic disease, the phenotypic variability in WD is considerable and the presence of a genotype-phenotype correlation has not yet been fully documented [12]. However, such cases have already been reported in the literature, analyzing siblings and monozygotic twins with WD. Chabik et al. highlighted the presence of a similar clinical and biochemical presentation of WD with a high intra-familial concordance, suggesting that analogue factors shared within the same families can strongly influence the disease presentation [13]; on the other hand, some authors have showed significant differences in phenotype despite the same genotype, assuming a significant influence on the WD phenotype by epigenetic/environmental factors [14–16]. A Chinese study had demonstrated a relationship between some pathogenetic variants and the different subtype and onset of WD. For example, the mutation *p.Arg919Gly* was related to neurological subtype and the *p.Arg778Leu* was related to younger onset age and lower levels of ceruloplasmin and serum copper. However, the research analyzed only the Chinese population, so more studies are needed for Caucasians WD patients [17]. Moreover, the incidence of this disease under certain issues appears to be closely related to so-called “founder effect”, that is the feature of certain “isolated” communities of contracting more frequently genetic autosomal recessive diseases, since mutations normally rare are more common in these communities [18]. In the present cases the diagnosis was made in according to clinical presentation, laboratory analyses including ceruloplasmin, serum and urinary copper 24 h levels, and radio-diagnostic investigations, with further diagnosis confirmation by genetic test for *ATP7B* gene.

The patients showed the similar set of genotype: compound heterozygosis for the mutations *c.3207C > A (p.His1069Gln) and c.3904-2A > G* of *ATP7B* gene*,* but their onset and clinical manifestation were different. The first pathogenetic variant found is a single nucleotide mutation in the 14th exon that causes the aminoacid substitution *of Histidine in position 1069 with Glutamine*. It appears to be the most frequent worldwide [12], with an allelic frequency from 17% in Italian population to 70% in Polish population among subjects affected by WD [19]. The second one, instead, is a splicing pathogenetic variant, disrupting the splice acceptor site at the 3′ end of intron 18–19, is reported with an allelic frequency of 0,276% in the United Kingdom in a cohort of WD patients [5]. These data confirm that WD shows a high genotypic heterogeneity, in which – with a few mutations of higher allelic frequency among affected subjects – there are many others of lower frequency. For this reason, it is more probable to find a number of patients with compound heterozygosis genotype higher than homozygosis genotype patients.

Despite the similar genotype, the onset of WD may be different: these sisters showed phenotypic differences at the onset and during the course of the disease involving different organs: case 1 with neurological symptom at onset (tremors, motor incoordination, dysarthria and dysgraphia) to portal systemic encephalopathy; case 2 with hepatic dysfunction started with hypertransaminasemia, hypertriglyceridemia and mild hepatomegaly to mild hepatic involvement.

At the follow-up, on brain MRI the case 1 always showed a worst clinical picture, with persistence of copper accumulation in basal ganglia. Moreover, she progressively developed hepatic cirrhosis and was put on the liver transplant list in association with a depressive symptomatology. The second one, instead, showed only a little liver involvement, with persistent mild hypertransaminasemia in absence of neuropsychiatric symptomatology. To note, both the patients showed a poor therapeutic compliance.

Phenotypic variability presented by these patients could be explained in different manners: firstly, the younger sister may not have developed neurological symptoms as WD was diagnosed 1 year earlier than the older sister (10 years old vs 11 years old). If the diagnosis would have been missed (unfortunately no data are available when she was asymptomatic), she may have developed neurologic symptoms a year later, as previous reported [12]. Secondly, the two sisters have different lifestyles: the elder one would have followed an irregular diet, with inadequate intake of nutrients, especially during adulthood. This may have favored the progression of WD and the different phenotype compared to the younger sister, who follows a Mediterranean-type diet, with reduced animal protein content. In support of this hypothesis, in literature is reported that a diet composed of limited animal protein intake could help to control hepatic copper stores [16]. Moreover, a recent study in animal model of WD indicates that the diet matters for the severity of the WD phenotype [20].

Finally, the intervention of modifier genes regulating copper metabolism in the presence of mutations *ATP7B,* as in WD, could be considered. Heterogeneous clinical presentations, probably, could not depend only on *ATP7B* degree of functional alterations or genetic mutation [21], but also it is presumable the action of pathogenic variants in other genes involved in copper homeostasis (defined in same studies as “*copper homeostasis genes*” or CHGs) [22], which could be analyzed in the future. Future research may change the therapeutic approach of WD patients in relation to their genotype, suggesting the necessity of a combined therapy where worst pathogenic variants or polymorphism of ATP7B and/or CGHs are present.

## Data Availability

The raw datasets generated and/or analysed during the current study are not publicly available in order to protect participant confidentiality.

## Abbreviations

ADHD: Attention Deficit Hyperactivity Disorder
ALT: ALanine aminoTransferase
AST: ASpartate Transaminase
CHGs: Copper Homeostasis Genes
CNS: Central Nervous System
CSF: Cerebrospinal fluid
Hb: Hemoglobin
KF: Kayser-Fleischer
LDH: Lactate dehydrogenase
MRI: Magnetic Resonance Imaging
PCE: PseudoCholinEsterase
PLT: Platelet
RBC: Red Blood Cells
WBC: White Blood Cells
WD: Wilson disease
